# Cytoplasmic Localization of RXRα Determines Outcome in Breast Cancer

**DOI:** 10.3390/cancers13153756

**Published:** 2021-07-26

**Authors:** Alaleh Zati zehni, Falk Batz, Vincent Cavaillès, Sophie Sixou, Till Kaltofen, Simon Keckstein, Helene Hildegard Heidegger, Nina Ditsch, Sven Mahner, Udo Jeschke, Theresa Vilsmaier

**Affiliations:** 1 Department of Obstetrics and Gynecology, University Hospital Munich LMU, 81377 Munich, Germany; Alaleh.Zati@med.uni-muenchen.de (A.Z.z.); falk.batz@med.uni-muenchen.de (F.B.); till.kaltofen@med.uni-muenchen.de (T.K.); simon.keckstein@med.uni-muenchen.de (S.K.); helene.heidegger@med.uni-muenchen.de (H.H.H.); Sven.Mahner@med.uni-muenchen.de (S.M.); Theresa.Vilsmaier@med.uni-muenchen.de (T.V.); 2 IRCM—Institut de Recherche en Cancérologie de Montpellier, INSERM U1194, Université Montpellier, Parc Euromédecine, 208 rue des Apothicaires, CEDEX 5, F-34298 Montpellier, France; vincent.cavailles@inserm.fr; 3 Faculté des Sciences Pharmaceutiques, Université Paul Sabatier Toulouse III, CEDEX 09, 31062 Toulouse, France; sophie.sixou@inserm.fr; 4 Cholesterol Metabolism and Therapeutic Innovations, Cancer Research Center of Toulouse (CRCT), UMR 1037, Université de Toulouse, CNRS, Inserm, UPS, 31037 Toulouse, France; 5 Department of Obstetrics and Gynecology, University Hospital, 86156 Augsburg, Germany; Nina.Ditsch@uk-augsburg.de

**Keywords:** breast cancer, retinoid X receptor, subcellular localization, steroid hormone receptor, prognosis, overall survival, disease-free survival, immunohistochemistry

## Abstract

**Simple Summary:**

Considering the immense development of today’s therapeutic approaches in oncology towards customized therapy, this study aimed to assess the prognostic value of nuclear versus cytoplasmic retinoid X receptor α (RXRα) expression in breast cancer. Our results demonstrate that RXRα expression may have different roles in tumorigenesis according to its subcellular localization. This study strengthens the need for further research on the behavior of RXRα, depending on its intracellular localization.

**Abstract:**

The aim of this retrospective study was to assess the prognostic value of cytoplasmic versus nuclear RXRα expression in breast cancer (BC) tissue samples and to correlate the results with clinicopathological parameters. In 319 BC patients, the expression of RXRα was evaluated via immunohistochemistry. Prognosis-determining aspects were calculated through uni- and multivariate analyses. Correlation analysis revealed a trend association with nuclear RXRα expression regarding an improved overall survival (OS) (*p* = 0.078), whereas cytoplasmic RXRα expression was significantly correlated with a poor outcomes in terms of both OS (*p* = 0.038) and disease-free survival (DFS) (*p* = 0.037). Strengthening these results, cytoplasmic RXRα was found to be an independent marker for DFS (*p* = 0.023), when adjusted to clinicopathological parameters, whereas nuclear RXRα expression was positively associated with lower TNM-staging, i.e., pT (*p* = 0.01), pN (*p* = 0.029) and pM (*p* = 0.001). Additionally, cytoplasmic RXRα expression was positively associated with a higher histopathological tumor grading (*p* = 0.02). Cytoplasmic RXRα was also found to be a negative prognosticator for Her-2neu-negative and triple-negative patients. Altogether, these findings support the hypothesis that the subcellular localization of RXRα plays an important role in carcinogenesis and the prognosis of BC. The expression of cytoplasmic RXRα is correlated with a more aggressive course of the disease, whereas nuclear RXRα expression appears to be a protective factor. These data may help to identify high-risk BC subgroups in order to find possible specific options in targeted tumor therapy.

## 1. Introduction

Breast cancer (BC) is the world’s most prevalent cancer and the most frequent cause of cancer death worldwide [1,2]. According to the World Health Organization (WHO) in 2020, 2.3 million women have been diagnosed with BC and 685,000 deaths were BC-related [3]. As a highly heterogeneous disease, BC diagnostics and treatment are complex and differ based on clinical tumor subtypes [4,5]. Options for BC therapies have advanced immensely over the past decades, offering a variety of therapeutic approaches depending on the therapy intention, i.e., in adjuvant, neoadjuvant or metastatic settings. Therapies include surgical interventions, radiation and systematic regimes such as chemotherapy and endocrine therapy [6,7,8]. Lately, novel therapeutic options have been introduced and implemented into international therapeutic guidelines in BC treatment, including, for instance, monoclonal antibodies targeting human epidermal growth factor receptor 2 (HER2). Therapies targeting nuclear receptors (NRs) such as the estrogen receptor (ER) and the progesterone receptor (PR) are very successful treatment options. Endocrine therapy regimes led to a decline in the BC-associated mortality rate of approximately 30%, which makes them indispensable for the treatment of hormone-receptor-positive (HR+) BC [9,10,11]. Furthermore, clinical trials indicate a strong correlation between the expression of “classical steroid hormone receptors”, such as ER and PR, and the progression of the disease [12,13,14,15,16]. Nevertheless, some tumors are resistant to those established therapeutic options, so the identification of new therapeutic targets is consequently central to research interests [17].

Currently, individual personalized BC therapy already entails NR-specific targeted therapies for both prevention and treatment [18]. NRs are activated by binding to lipophilic metabolites and mainly function as transcription factors in the nucleus [19,20]. Novel literature demonstrates that, besides the well-established NR, other receptors, including the retinoid X receptor (RXR), thyroid hormone receptors (THRs) and vitamin D receptor (VDR), play a significant role in the pathophysiology not only of BC but also of other cancer entities [21,22,23]. Studies on the role of NR in distinct intracellular compartments indicate a specific prognostic value, depending on the subcellular localization [24]. Czogalla et al. demonstrated a direct link between the cytoplasmic localization of VDR and impaired overall survival (OS) in ovarian cancer [25]. In the case of THR, high nuclear localization was reported to be a positive predictive factor for OS in epithelial ovarian cancer [22]. In contrast, nuclear THR has been identified to have cancer-promoting activities in BC development [24].

As a promising protective and antitumoral factor, RXRs appear to be regulatory factors with important roles in various processes during the initiation and progression of BC [26]. RXRα plays an important role in innate and adaptive immune responses by coordinating cell metabolism and the mononuclear phagocyte system [27,28]. Previous studies revealed that the activation of RXRα regulates levels of cytokines and chemokines, coordinates phagocytosis after apoptosis and attenuates antiviral immune reactions in myelocytes [27,29]. RXRα controls cell differentiation, is a crucial factor for physiological cell homeostasis and operates as a potent regulator of pathogenesis in diverse diseases, including BC [30,31]. Retinoids derived from vitamin A and co-activator molecules bind and activate RXRs, which then regulate the transcriptional activity by means of heterodimers with other nuclear receptors such as THR and VDR and translocates into the nucleus to eventually promote its transcriptional activity [31]. Since retinoids have been defined as inductors of cancer cell differentiation and cell proliferation arrest, they are key components in the tumorigenesis process and cancer cell metabolism [20,32]. After activation and heterodimerization, RXR subsequently translocate from the cytoplasm to the nucleus [33] and binds to promoters of the target genes [34]. So far, three different RXRs have been described: RXRα (NR2B1), RXRβ (NR2B2) and RXRγ (NR2B3) [35].

To date, specific pathways, such as the nuclear import of RXR-VDR heterodimers, have been investigated. Yet the exact mechanisms that determine the translocation of RXR-containing heterodimers into the nucleus remain insufficiently described [36]. Zhang et al. reported that RXRα modulated important biological processes in the cytoplasm, such as mitochondria-dependent apoptosis, inflammation and phosphatidylinositol 3-kinase (PI3K)/AKT-mediated cell survival [37]. Interestingly, new small-molecule-binding sites on the surface of RXRα have been identified recently, which are described to mediate the regulation of the nongenomic actions of RXRα by means of a class of small molecules derived from a nonsteroidal anti-inflammatory drug [37]. RXRα expression is described to be increased in miscarriage in the endometrial glands, a process linked to inflammation [38]. RXR consists of an amino-terminal domain, which plays a role in the basal transcriptional activity, a zinc finger containing a DNA binding domain, a connecting hinge region and a carboxyl-terminal region (ligand binding domain, LBD). The LBD binds the ligand, regulates interactions with transcriptional co-regulators and coordinates the formation of dimers or (in the case of RXR) of tetramers. Thus, the LBD regulates the ligand-dependent transcriptional activities [39]. In the absence of a ligand, RXRs are transcriptionally silent. After being activated by its ligand, RXRs form homotetramers, which dissociate immediately, or RXRs heterodimerize with other nuclear receptors such as VDR and initiate transcriptional activity [39,40]. Since RXR-containing oligomers play a significant role in the nucleus, it is crucial to understand the mechanisms that underlie the nuclear translocation that for now have not been investigated sufficiently. According to the Human Protein Atlas (https://www.proteinatlas.org/ENSG00000186350-RXRA/tissue/breast, accessed on 19 July 2021) there is an intense expression of RXRα exclusively in the nucleus of normal glandular breast tissue and 40% of these cells show a positive RXRA expression in premenopausal women, and 15% show positive expression in postmenopausal women [41].

In cell studies, Raffo et al. identified RXRs as specific mediators of proliferation inhibition and apoptosis induction in BC cell lines [42]. Furthermore, in comparison with benign breast tissue, Friedrich et al. found higher expression levels of RXR in breast cancer cells [43]. As suppressors of cell proliferation, these receptors inhibit tumor cell growth [32]. Studies on BC cell lines and in vivo approaches demonstrated a proapoptotic effect of RXR in BC cells, which is likely to harm BC growth [44,45,46]. Moreover, there is evidence of a cooperative interaction between RXR and ER in this antiproliferative process [47,48]. Several studies suggest that RXR might represent a marker or even a therapeutic target in BC. Furthermore, the literature indicates that high expression of RXR is an antitumoral mediator and thus a favorable prognostic factor in BC [49,50,51]. On the contrary, our previous study identified RXR expression as a risk factor with a significant worse DFS in patients with multifocal or multicentric BC [52].

Due to its alleged contradictory role in BC prognosis, it appeared necessary to further investigate the behavior of RXR in BC. No study so far has identified the subcellular localization of RXRα as a prognostic factor in human breast cancer specimens. New insights could potentially be promising in regard to individualized targeted BC therapy. In the present study, we define the prognostic role of cytoplasmic versus nuclear expression of RXRα in BC and correlate the results with clinicopathological criteria.

## 2. Materials and Methods

### 2.1. Patient Collective

The cohort for this study includes 319 formalin-fixed paraffin-embedded primary BC tissues that were collected from patients who underwent surgery from the year 2000 to 2002 at the Department of Gynecology and Obstetrics of the Ludwig Maximillian University in Munich, Germany.

The total collective (TC) included 319 patients (Table 1). After an observation period of up to 10 years, DFS and OS were statistically analyzed, and these follow-up data were retrieved from the Munich Cancer Registry. The Union for International Cancer Control (UICC) TNM classification was completed to assess the size of the primary tumor (pT) [53,54], lymph node involvement (pN), and distant metastasis (pM). An experienced pathologist of the LMU Department of Pathology determined the tumor grade and histological status. Tumor grade was defined according to the Bloom and Richardson grading system [55]. The hormone receptor status was determined by immunohistochemistry on paraffin-embedded material. Cells were regarded as hormone-receptor-positive with positive staining in ≥10% of the tumor cell nuclei. The immune-reactive scoring system (IRS) of Remmele and Stegner (IRS) was used [56].

### 2.2. Patient Treatment

As described previously [57,58], the primary surgical treatment involved either breast conservation or modified radical mastectomy. Routine axillary dissections were performed on level I and II lymph nodes, whereas level III lymph nodes were only excised in the expression of macroscopic metastatic lesions of the lower levels was observed. Single embedded lymph nodes were screened at up to three levels for the diagnosis of lymph node metastasis.

According to the guidelines of the Cancer Treatment Center of Munich, the patients in this study received chemotherapy in cases of lymph node involvement. Hormone receptor-positive postmenopausal patients received adjuvant endocrine therapy with tamoxifen (20–30 mg/day). Premenopausal woman received GnRH analogues in the later years of the follow-up period. Aromatase inhibitors were used in the case of contraindications.

However, the guidelines for surgical, radiation oncology and chemotherapy treatment options changed substantially within the observation time of the study. Therefore, the authors did not include oncological treatment details. Nevertheless, in our patient cohort, the most common type of chemotherapy consisted of six cycles of CMF, cyclophosphamide (600 mg/m^2^ body-surface area), methotrexate (40 mg/m^2^) and 5-fluoruracil (600 mg/m^2^) every 21 days.

### 2.3. Immunohistochemistry

According to the earlier published and well described methods [52,59,60,61], the immunohistochemistry of RXRα on formalin fixed paraffin-embedded sections was assessed. Concisely, a combination of pressure cooker heating and the standard streptavidin-biotin-peroxidase complex with a mouse/rabbit-IgG-Vectastain Elite ABC kit (Vector Laboratories, Burlingame, CA, USA) were used. The staining procedure was carried out with commercially available kits with a monoclonal mouse antibody to detect RXRα expression (Perseus Proteomics Inc., Tokyo, Japan).

Therefore, the paraffin-embedded tissue sections were dewaxed in xylol for 15 min and rehydrated twice for 15 min in a solution containing 100% alcohol. Endogenous peroxidase activity was quenched by immersion in 3% hydrogen peroxide (H_2_O_2_) (Merck; Darmstadt, Germany) in methanol for 20 min. Again, sections were put into a 96% and then a 70% alcohol solution. After washing in phosphate-buffered saline (PBS), sections were subjected to 10 min in a pressure cooker using sodium citrate buffer with a pH of 6.0 for epitope retrieval. To create the pH of 6.0, 0.1 citric acid was diluted in 1 L distilled water (solution A) and 0.1 M sodium citrate was diluted in 1 L distilled water (solution B). The solution contained 18 mL of solution A and 82 mL of solution B, diluted with 900 mL distilled water. The steps involved in washing the sections in distilled water and PBS were then carried out. To prevent the nonspecific binding of the primary antibodies (Anti-RXRα), sections were incubated with diluted normal serum (10 mL PBS containing 150 μL horse serum, Vector Laboratories). Afterwards, incubation of the tissue sections with the primary antibodies diluted in PBS (1:1000) was carried out for one hour at room temperature. Sections were washed twice for 2 min in PBS. Incubation with the secondary antibody binding the streptavidin-biotin-peroxidase complex (ABC-Complex), diluted in 10 mL PBS for 30 min, was performed. This was followed by repeated PBS washing steps and incubation with the ABC complex. Coloration of the substrate was achieved with the use of chromogen 3,3′-diaminobenzidine (DAB; Dako, Glostrup, Denmark) for one minute. After washing in PBS, sections were counterstained with Mayer’s acidic hematoxylin for 2 min. Finally, the sections were rehydrated in an ascending alcohol series and covered with Eukitt.

**Figure 1:**
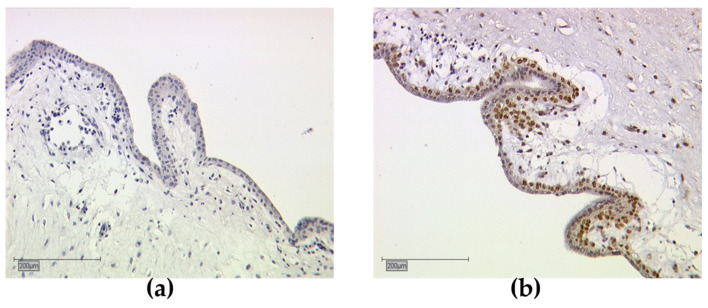
Immunohistochemical staining of retinoid X receptor (RXR) in human placenta tissue, serving as negative and positive control; (**a**) RXRα-negative control and (**b**) positive control. (**a**,**b**) shows 10× (scale bar = 200 µm) magnification.

Serving for negative controls were human placenta tissue sections incubated with pre-immune IgGs (supersensitive rabbit negative control, BioGenex, Fremont, CA, USA), instead of the primary antibody. As positive controls, we used human placenta samples for RXRα detection (Figure 1). Pictures were taken with a digital charged coupled device (CCD) camera system (JVC, Tokyo, Japan).

### 2.4. Staining Evaluation (Immunoreactive Score)

To quantify the specific RXRα immunoreactivity in the nuclei and the cytoplasm, meaning the distribution and intensity patterns, the well-established semi-quantitative immune-reactive scoring system (IRS) of Remmele and Stegner (IRS) [56] was used. Two independent blinded observers evaluated the intensity and distribution pattern of the staining reaction. In six circumstances (*n* = 1.9%), the assessment of the two independent observers varied. Both observers re-evaluated these cases together, eventually agreeing upon the same result. The concordance prior to the re-evaluation was stated at 98.1%. The scoring method has previously been described and used in numerous studies conducted by our study group [52,59,60,61]. Analyzing the staining, a light microscope by Leitz (Immunohistochemistry Type 307–148.001 512 686) (Wetzlar, Germany) and a 3CCD color camera (JVC, Victor company of Japan, Japan) were used.

The IRS scoring system ranges from 0 to 12. To obtain the IR score results, the staining intensity (score 0 = no staining, score 1 = weak staining, score 2 = moderate staining, score 3 = strong staining) and the percentage of positively stained cells (0: no staining, 1: ≤10% of the cells, 2: 11–50% of the cells, 3: 51–80% of the cells and 4: ≥81% of the cells) were multiplied.

Nuclear and cytoplasmic stainings of RXRα were evaluated in parallel, with the separate determination of nuclear and cytoplasmic IRS. Cut-off scores for the IRS were defined as follows: tissue samples that had been assigned an IRS greater than 1, for either nuclear or cytoplasmic RXRα expression, were scored as positive.

### 2.5. Ethical Approval

The tissue samples used in this study were left over material after all diagnostics had been completed and were retrieved from the archive of Gynecology and Obstetrics, Ludwig Maximilian University, Munich, Germany. All patients gave their consent to participate in the study. All patient data and clinical information from the Munich Cancer Registry were fully anonymized and encoded for statistical analysis. The study was performed according to the standards set in the Declaration of Helsinki, 1975. The current study was approved by the Ethics Committee of Ludwig Maximilian University, Munich, Germany (approval number 048–08). The authors were blinded from the clinical information during the experimental analysis.

### 2.6. Statistical Analysis

Statistical analysis was performed with the IBM Statistical Package for the Social Sciences (IBM SPSS Statistic v26.0 Inc., Chicago, IL, USA). The gathered results were inserted into the SPSS database in the implied manner, building the TC. The Chi-squared test was used to assess the distribution of clinical-pathological variables. Correlations between findings of immunohistochemical staining were determined with Spearman’s analysis. The nonparametric Kruskal–Wallis test was used to test for differences in cytoplasmic and nuclear RXRα expression regarding the set prognostic markers. OS (in years) and disease-free survival (DFS) (in years) was compared using Kaplan–Meier graphics and differences in patient survival times were tested for significance using the Chi-squared statistics of the log rank test. For multivariate analyses, the Cox regression model for survival was used and the following factors were included: pT and pN of the TNM staging system, grading, histology type, focality, and estrogen and progesterone receptors. Each parameter to be considered showed significance at the level of *p* < 0.05. The *p*-value and the number of patients analyzed in each group are given for each chart.

## 3. Results

### 3.1. Patient Characteristics

Table 1 presents a summary of the detailed patient characteristics from the TC. The relatively large TC, including 319 patients, and the fairly equal distribution amongst the subgroups, strengthen the statistical power of our study. The mean age (±STDV) of the cohort at the time of initial diagnosis was 59 ± 13.1 years. In terms of BC focality, 173 patients were diagnosed with unifocal and 146 with multifocal and/or multicentric BC. A total of 61.4% of the patient collective had histological invasive carcinoma of no special type (NST); 81.7% of BC patients were ER-positive, whereas 62.6% of patients were PR-positive; 52.2% of patients had a low-grade carcinoma (G1–G2 = 52.2%, G3 = 47.7%) and 64.3% were staged with a tumor size smaller than 2 cm (pT1: 64.3%, pT2–pT4: 35.6). Furthermore, 54.2% of all patients were staged pN0. The majority of the patient collective was staged with no present metastasis at initial diagnosis (pM0 = 78.1%, pM1 = 21.8%). A total of 12 patients showed a high cytoplasmic expression and 37 patients showed a high nuclear expression. The cut-off for high expression was IRS scores > 4. The negligible different total patient numbers (*n*) in the subgroups may be explained by the lack of a limited number of input variables, which could not be obtained due to the retrospective character of the study.

### 3.2. RXRα Expression Correlates with Clinicopathological Data

**Figure 2:**
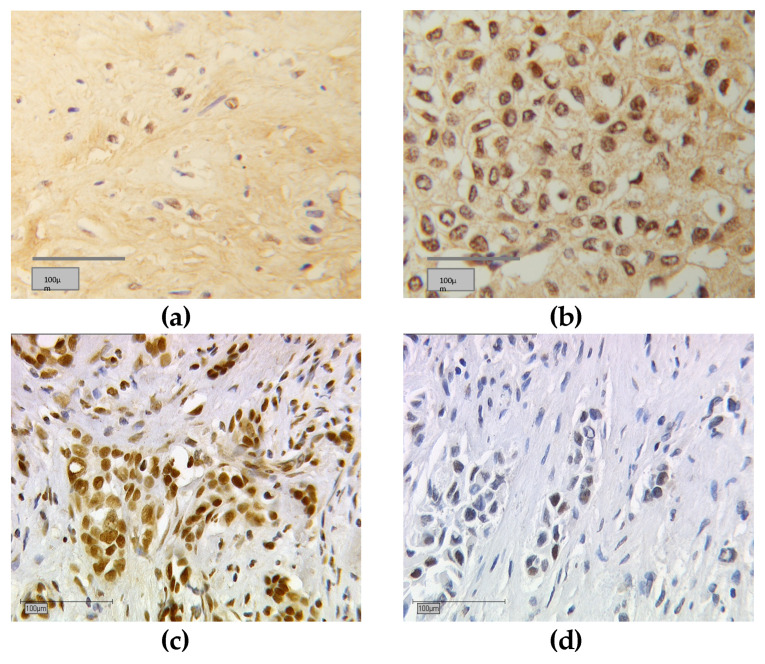
Immunohistochemical staining of retinoid X receptor (RXR). Immunohistochemical staining of RXRα in human breast cancer samples is illustrated in (**a**–**d**): (**a**) positive cytoplasmic RXRα expression only, (**b**) positive nuclear and cytoplasmic RXRα expression with a nucleo-cytoplasmic IRS ratio of 8:4, (**c**) positive nuclear RXRα expression only, (**d**) negative nuclear and cytoplasmic RXRα expression; (**a**–**d**) show 25× (scale bar = 100 µm) magnification.

The distribution of tumors, with negative or positive nuclear or cytoplasmic RXRα staining, was analyzed for all tumors. Figure 2 shows immunohistochemically stained RXRα images. Positive stained tissue appeared in a brownish color (Figure 2a–c) and negative or unstained cells appeared blue (Figure 2d). Human placenta tissue was used for RXRα negative controls (Figure 1a) and positive controls (Figure 1b). Tumor staining either appeared negative or positive for both nuclear and cytoplasmic RXRα localizations (Table 1). In cases of RXRα expression in both localizations, a nucleo-cytoplasmic IRS ratio was given (Figure 1b). Correlation analyses show the results of any nuclear or cytoplasmic staining present.

RXRα expression displayed correlations to clinical and pathological data (Table 2) in univariate analysis, using the Spearman correlation coefficient. A positive correlation was observed between high cytoplasmic RXRα expression and higher histopathological tumor grading (*p* = 0.029; *Cc* = 0.125). In the nucleus, RXRα was significantly and negatively associated with tumor size (pT) (*p* = 0.012; *Cc* = −0.143).

In addition, Kruskal–Wallis analysis revealed a statistical positive association for nuclear RXRα expression in BC tissue samples with lower pN (*p* = 0.029) and lower pM (*p* = 0.001) staging cases in BC patients. No further significant association between nuclear or cytoplasmic RXRα expression and clinicopathological characteristics could be found.

### 3.3. Nuclear RXRα Expression Correlates with Clinicopathological Parameters Linked with Good Prognosis

**Figure 3:**
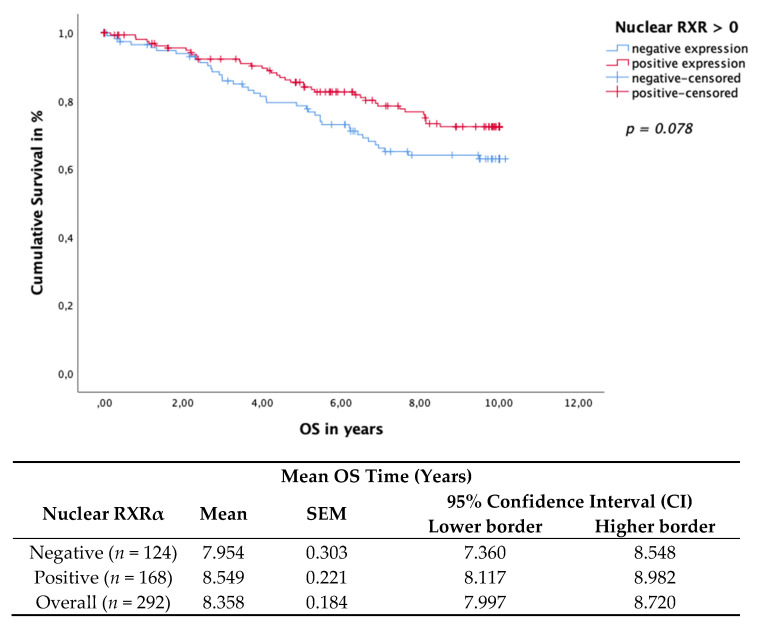
Kaplan–Meier survival analysis of nuclear RXRα positive and negative expression in relation to OS. The risk table demonstrates the mean survival time, SEM and 95% confidence interval (CI) for univariate analyses.

Nuclear RXRα expression in BC tissue samples revealed a trend association with improved OS. The Kaplan–Meier curve visualized a positive association of OS (Figure 3) with the expression of nuclear RXRα. The log rank test yielded a *p*-value of 0.078 for the OS, which was not statistically significant per our definition. Nevertheless, an obvious trend in terms of a positive correlation of nuclear RXRα expression with OS can be observed. Nuclear RXRα expression showed no significant effect on the DFS (*p* = 0.914), calculated using the Kaplan–Meier curve and the log rank test. Finally, multivariate Cox regression identified nuclear RXRα as a dependent prognostic factor for OS (HR 0.733, 95%CI 0.487−1.103, *p* = 0.136) (Table 3).

### 3.4. Cytoplasmic RXRα Is an Independent Prognostic Factor for DFS

**Figure 4:**
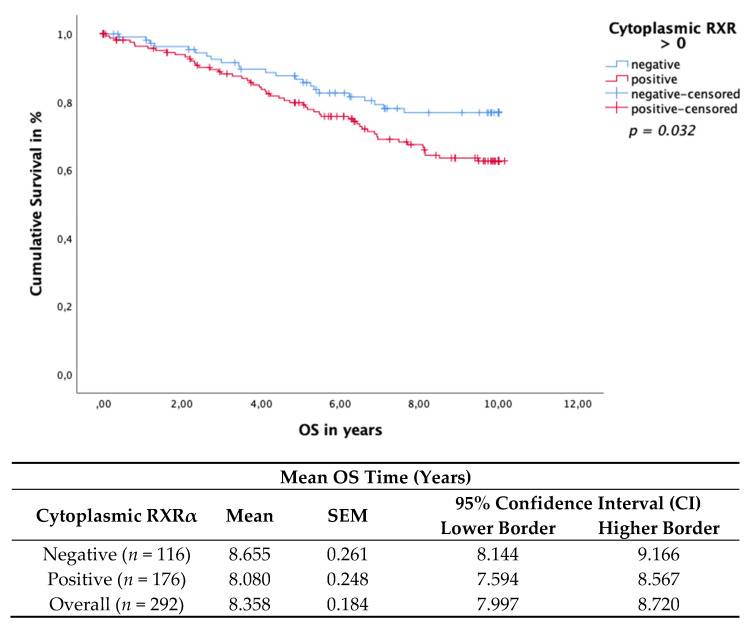
Kaplan–Meier survival analysis of cytoplasmic RXRα positive and negative expression in relation to OS. Statistical significance is shown as *p*-value determined using the log-rank-test (*p* < 0.05). The risk table demonstrates the mean survival time, SEM and 95% confidence interval (CI) for univariate analyses.

**Figure 5:**
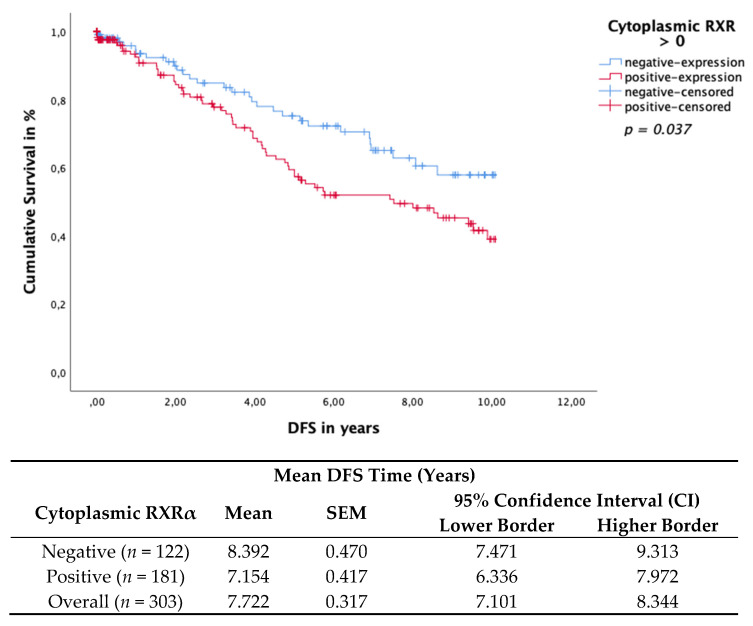
Kaplan–Meier survival analysis of cytoplasmic RXRα positive and negative expression in relation to DFS. Statistical significance is shown as *p*-values determined using the log-rank-test (*p* < 0.05). The risk table demonstrates the mean survival time, SEM and 95% confidence interval (CI) for univariate analyses.

Cytoplasmic RXRα expression in BC tissue samples was associated with impaired OS and DFS. The Kaplan–Meier curve visualized a significant negative association of the OS (Figure 4) and DFS (Figure 5) when expressing cytoplasmic RXRα. A statistically negative significant correlation was observed for the OS (*p* = 0.032) and for the DFS (*p* = 0.037), as calculated by the log rank test.

Multivariate Cox regression identified cytoplasmic RXRα as a dependent prognostic factor for OS (HR 1.603, 95%CI 0.964–2.665, *p* = 0.069) (Table 4) but an independent factor for DFS (HR 1.696, 95%CI 1.077–2.671, *p* = 0.023) (Table 5).

### 3.5. Subcellular Localisation of RXRα

Breast cancer cases were furthermore classified into four combinatorial phenotypic groups as follows: (1) cytoplasmic and nuclear RXRα expression negative (*n* = 47); (2) cytoplasmic RXRα expression positive only (*n* = 83); (3) nuclear RXRα expression positive only (*n* = 75); (4) cytoplasmic and nuclear RXRα expression positive (*n* = 98). As described previously, cut-off scores for the IRS were defined as follows: tissue samples that had been assigned an IRS greater than 1, for either nuclear or cytoplasmic RXRα expression, were scored as positive. Tumor staining results appeared either negative or positive for both nuclear and cytoplasmic RXRα localizations. In cases of RXRα expression in both localizations, a nucleo-cytoplasmic IRS ratio was given.

In the patient cohort with a positive nuclear RXRα expression only, this group revealed a significant correlation with improved OS and DFS when compared to the other combinatorial RXRα expression cohorts.

**Figure 6:**
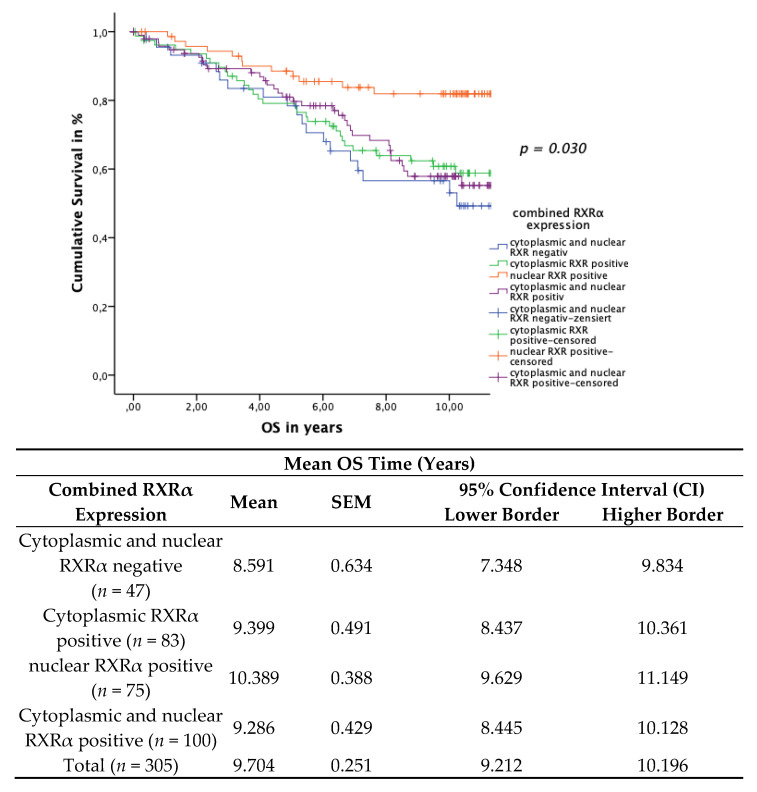
Kaplan–Meier survival analysis of the four combinatorial phenotypic RXRα groups and their expression in relation to OS. Statistical significance is shown as *p*-values determined using the log-rank-test (*p* < 0.05). The risk table demonstrates the mean survival time, SEM and 95% confidence interval (CI) for univariate analyses.

**Figure 7:**
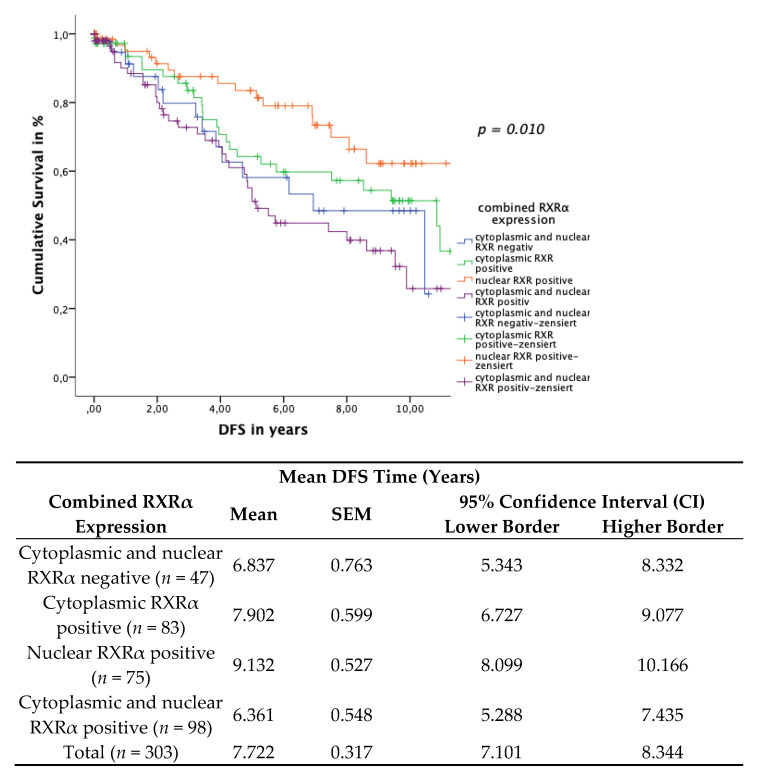
Kaplan–Meier survival analysis of the four combinatorial phenotypic RXRα groups and their expression in relation to DFS. Statistical significance is shown as *p*-values determined using the log-rank-test (*p* < 0.05). The risk table demonstrates the mean survival time, SEM and 95% confidence interval (CI) for univariate analyses.

The Kaplan–Meier curve visualized a positive correlation of OS (Figure 6) and a positive correlation of DFS (Figure 7) with the expression of nuclear RXRα only in contrast to the combinatorial RXRα expression groups. The log rank test yielded a significant correlation both for OS (*p* = 0.030) and for DFS (*p* = 0.010).

### 3.6. Cytoplasmic RXRα Is a Negative Prognosticator for Her-2neu-Negative and Triple-Negative Patients

Breast cancer cases were furthermore divided into the known subgroups; ER, PR, Her-2neu and triple-negative cases. The prognostic value of RXRα expression in the different subcohorts was analyzed and revealed a significant correlation in Her-2neu negative and triple-negative patients and RXRα expression. No further BC subtype group displayed a prognostic association with RXRα expression.

**Figure 8:**
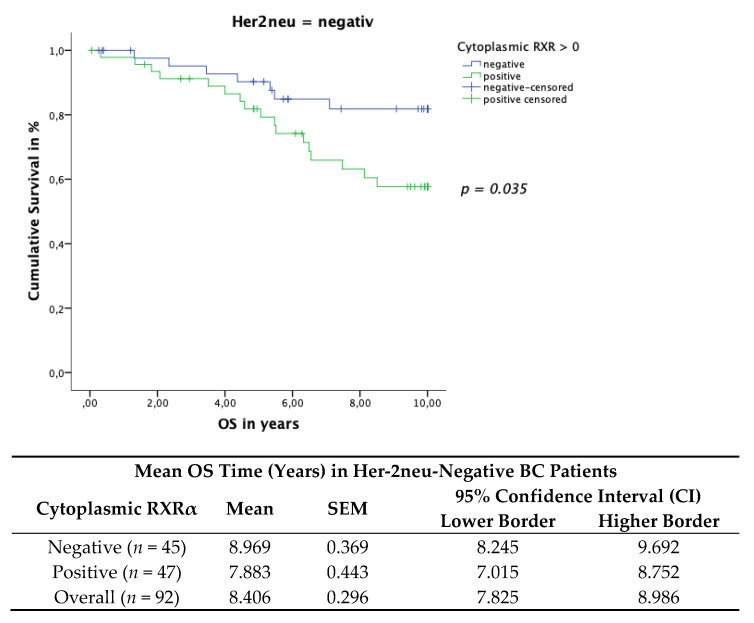
Kaplan–Meier survival analysis assessing cytoplasmic RXRα-positive expression in the Her-2neu-negative patient cohort in relation to OS. Statistical significance is shown as *p*-values determined using the log-rank-test (*p* < 0.05). The risk table demonstrates the mean survival.

The Her-2neu-negative patient cohort with positive cytoplasmic RXRα BC tissue expression revealed a significant correlation with impaired OS. The TC included 94 patients with a Her-2neu-negative status and 95 patients with a Her-2neu-positive status. The Kaplan–Meier curve visualized a negative correlation of the OS (Figure 8) with the expression of cytoplasmic RXRα in Her-2neu-negative patients. The log rank test yielded a significant correlation for the OS (*p* = 0.035). Her-2neu-positive patients revealed no significant correlation in relation to survival (*p* = 0.471). Her-2neu-positive or negative patients revealed no statistical correlation in relation to nuclear RXRα-positive (*p* = 0.415) or negative (*p* = 0.351) expression and survival.

**Figure 9:**
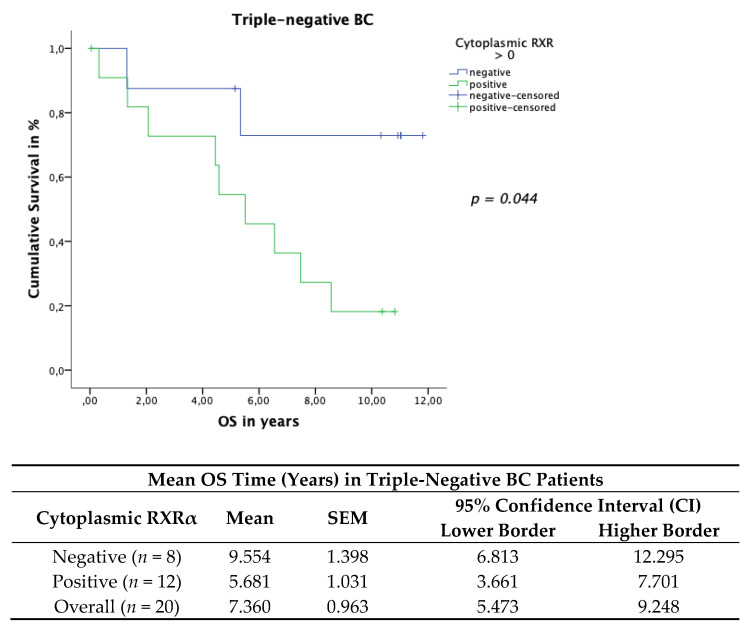
Kaplan–Meier survival analysis assessing cytoplasmic RXRα positive expression in the triple-negative patient cohort in relation to OS. Statistical significance is shown as *p*-values determined using the log-rank test (*p* < 0.05). The risk table demonstrates the mean survival time in triple-negative patients, SEM and the 95% confidence interval (CI) for univariate analyses.

The triple-negative patient cohort with a positive cytoplasmic RXRα BC tissue expression revealed a significant correlation with worse OS. The TC included 20 patients with a triple-negative BC status. The Kaplan–Meier curve visualized a negative correlation of the OS (Figure 9) with the expression of cytoplasmic RXRα in triple-negative patients. The log rank test yielded a significant correlation for the OS (*p* = 0.044).

## 4. Discussion

The aim of this study was to evaluate the prognostic impact of the subcellular expression of RXRα in a large cohort of BC tissues, and to correlate the results with clinicopathological criteria. To date, the role of RXRs in BC patients has not been sufficiently investigated [32]. This is the first study to define the prognostic role of cytoplasmic versus nuclear expression of RXRα in BC using a relatively large patient cohort that did not receive any treatment before surgery and a long-term follow-up. Results from the present study provide evidence that the expression of cytoplasmic RXRα is a significant negative prognostic marker, whereas nuclear RXRα expression appears to be a protective factor.

To understand better the molecular function of RXRα in the pathogenesis of BC, this study focused separately on nuclear versus cytoplasmic RXRα expression in BC. Our study confirmed that RXRα is expressed with a nuclear and cytoplasmic localization. Interestingly, nuclear and cytoplasmic forms of RXRα may exhibit opposite roles in mammary carcinogenesis. This is possibly due to the activation status of RXR. Inactivated cytoplasmic RXRα does not show anticancerogenic potential, in contrast to the activated nuclear form of RXRα. Furthermore, the presence of inactive cytoplasmic RXRα may also be due to the absence of a corresponding ligand or decreased receptor response to the ligand. This could cause lower levels of activated nuclear RXRα. Indeed, considering the correlation with the cytoplasmic expression of RXRα, DFS and OS were significantly lower, whereas the nuclear expression of RXRα revealed a trend association with improved OS. Similarly, the patient cohort with positive nuclear RXRα expression only revealed a significant correlation with improved OS and DFS when compared to the other combinatorial RXRα expression cohorts. These correlations are strengthened by the fact that, in the multivariate analysis, cytoplasmic RXRα was found to be an independent prognostic marker for poorer outcomes in DFS and a dependent prognostic factor in OS. However, nuclear RXRα was found to be a dependent prognostic marker for DFS and OS.

The role of RXRα in DNA binding and transactivation is known; however, several studies indicate that RXRα also has extranuclear functions. RXRα is reported to reside in the cytoplasm at different stages of development and can migrate from the nucleus to the cytoplasm in response to differentiation, survival, apoptosis and inflammation [37,62,63,64,65]. In our study, cytoplasmic RXRα in BC tissues was negatively associated with patient survival, whereas nuclear RXRα expression appeared to be a protective factor. Numerous studies have confirmed that modification of the subcellular localization of RXRα is connected to the development of malignant diseases and inflammation processes, thus offering potential explanations for this difference at the cellular level [37]. Ghose et al. described how inflammation processes reduced the nuclear localization of RXRα in the liver and how inflammation-induced signaling can lead to fast, diverse and multiple alterations in hepatic gene expression [62]. Mey et al. confirmed RXRα translocation from the nucleus to the cytoplasm in response to endotoxin and other inflammatory mediators and described how it inhibits its transactivation function [63]. In highly malignant human breast cancer cells, an altered localization of RXRα to the splicing factor compartments was confirmed [64], whereas Zhou et al. reported that an N-terminally truncated form of RXRα produced in cancer cells resides in the cytoplasm to promote tumor cell growth [65].

To date, no studies have described the specific role of RXRα in different cell compartments in BC tissue. However, recent data on other nuclear receptors in cancer research indicate that the prognostic value of nuclear receptors depends on their subcellular localization [24]. Consistently with our findings, Ditsch et al. demonstrated that there is a direct link between the nuclear localization of THR and increased OS in epithelial ovarian cancer [22]. However, THR has been identified to show cancer-promoting activities during BC development [24]. In the case of VDR, a direct link between the cytoplasmic localization of VDR and impaired OS in ovarian cancer has been reported [25]. Furthermore, higher cytoplasmic VDR expression in colon and vulvar cancer, as well as in malignant melanoma, had an impact on tumor progression and prognosis [66,67,68].

In order to gain further insights on potential individualized targeted BC treatments, we evaluated the subcellular RXRα expression in correlation with clinicopathological characteristics. In our study, cytoplasmic RXRα expression was strongly positively correlated with higher histopathological tumor grading. Nuclear RXRα expression, on the other hand, was significantly positively associated with a decreased tumor size, as well as lower pathological staging for distant metastases and regional lymph node involvement. Our data suggest a specific role of the subcellular localization of RXRα. In this study, cytoplasmic RXRα was significantly correlated with a worse prognosis in BC. Furthermore, a trend towards favorable prognostic outcomes could be seen in the case of nuclear RXRα in BC tissue. Interestingly, when looking at the different BC subcohorts in our patient collective, triple-negative and Her-2neu-negative BC tissue that stained positive for cytoplasmic RXRα showed a significant decrease in OS. These results further support the hypothesis that the shuttling of RXRα out of the nucleus, or RXRα knock-out, may lead to a deterioration of survival. No studies have so far examined the prognostic value of the intracellular expression of RXRα in BC subcohorts. Previously, Joseph et al. reported that high-nuclear retinoid X receptor gamma (RXRɣ) expression in ER-positive BC tissue samples was associated with a better prognostic impact [69]. This is consistent with previous reports indicating a positive prognostic value of nuclear RXRɣ in ER- and PR-positive BC [70]. Nevertheless, the mechanisms by which the intracellular localization of RXRs exert their effects in BC subtypes remain incompletely understood [32]. The more detailed investigation of the intracellular localization of the RXR protein in BC in triple-negative breast cancer is of particular interest, as this BC subtype is characterized by worse OS, DFS and increased metastatic potential compared with other major BC subtypes. The identification of reliable predictive biomarkers is fundamental in finding new therapeutic regimes.

In cancer research, RXR has been found to be a rather protective factor. Lee et al. documented impaired OS in the case of the epigenetic inactivation of RXR genes in non-small cell lung cancer [71]. Furthermore, both the activation of RXR and the higher expression of RXR in epithelial ovarian cancer have been found to foster pro-apoptotic mechanisms [72]. In BC cells and other tumor entities, RXR is able to execute responses such as cell proliferation, cellular differentiation and programmed cell death [45,69]. Consequently, RXR is a potential therapeutic target in breast cancer cell lines [44,45]. Earlier research indicates the high expression of RXR to be an antitumoral mediator und thus a favorable prognostic factor in BC [49,50,51]. Consistently, cell studies on BC cells demonstrated upregulated apoptosis in BCL2-positive human cancer cells after the activation of RXR [44].

Furthermore, in vivo studies on transgenic mice demonstrated that RXR ligands decreased vascularization in BC [73]. Additionally, Wu et al. indicated that RXR-selective retinoids in BC tumors in transgenic mice prevented carcinogenesis and that receptor-selective retinoids may be an option in the molecular-based chemoprevention of BC [74]. For instance, LGD1069, an RXR-selective retinoid, was described to be a promising therapeutic agent. After the binding and activation of RXR, LGD1069 leads to a downregulation of cyclooxygenase-2 expression and induces a temporary G1 cell cycle arrest in human breast cells [75,76]. In relation to hereditary breast cancer, RXR has been described to be overexpressed in BRCA1-mutated BC cells. Consequently, RXR may potentially serve as a marker or even a therapeutic target in hereditary breast cancer [49].

In contrast to previous findings, which attribute a protective and antineoplastic function to RXR, our previous study identified RXR expression to be a risk factor, correlated with a significantly worse DFS in patients with multifocal or multicentric BC [52]. To explain the tumorigenic function of RXR in multifocal or multicentric BC, we suggest an increased interaction between RXR and other NRs such as VDR with elevated levels of heterodimers. Ditsch et al. previously identified RXR, THR and VDR to form functional homodimers and heterodimers with many other NRs in human BC cell lines [31]. Depending on their activation status and possibly their corresponding NRs, specific responses such as growth arrest and apoptosis may be induced. Thus, we assume a protective role of these NRs in breast cancer development [45]. Contrary to this assumption, a multicenter phase II study of oral bexarotene, a third-generation retinoid which is indicated for the treatment of cutaneous T-cell lymphoma, did not show an inhibitory effect on cancer growth. This therapeutic approach was ineffective for patients with metastatic BC [77,78]. Due to the abovementioned contradictory data on the role of RXR in BC development and progression, further scientific research on RXR is needed.

There are some factors limiting our study. First, it was a retrospective analysis based on a single dataset. Second, the sample size was comparatively low and may thus be possibly insufficient to elucidate all the heterogenous entities in breast cancer. Furthermore, specific information on possible toxic environmental aspects or a history of endocrine therapy and other patient characteristics could enrich the investigation of how additional factors interact with RXRα. Hormone treatment with estrogen might influence breast cancer growth, since a cooperative antiproliferative interaction between RXR and ER has been described [47,48]. In addition, the guidelines for surgical, radiation oncology and chemotherapy treatment options changed substantially within the observation time of the study. Therefore, the authors did not include oncological treatment details.

However, aside from these limitations, our data demonstrate that the RXRα pathway could represent a promising therapeutic target in BC. This study might provide an impetus to further investigate the crosstalk between potential NR ligands and the RXRα pathway in regard to its therapeutic potential in BC.

In summary, our findings demonstrate the complexity of the links between nuclear and cytoplasmic RXR expression and their impact on patient outcome. Our results emphasize the need for more detailed investigations of the intracellular localization of the RXR protein in mammary carcinoma in order to understand its biomolecular function and role as a possible biomarker in BC diagnostics.

## 5. Conclusions

In this study, we investigated the prognostic value of the nuclear localization of the RXRα receptor versus its cytoplasmic expression in human BC specimens. Furthermore, we investigated the correlation between clinicopathological criteria as well as patient outcomes and the subcellular localization of RXRα. This is the first retrospective cohort study to define the prognostic role of cytoplasmic versus nuclear expression of RXRα in sporadic mammary cancer using a large clinical patient cohort and a long-term follow-up. RXRα expression was observed to play an incongruous role for BC prognosis depending on its intracellular localization: RXRα expressed in the cytoplasm of BC tissues was negatively associated with prognostic factors, such as patient survival, and the opposite result was observed in nucleus-localized RXRα.

In summary, nuclear receptors, such as RXRα, and possible targeted treatments should become the subjects of future research. Our findings confirm the need of further examinations on the subcellular expression of RXRα and other members of the NR family. Further investigations studying the biomolecular role of RXRα in BC would be of major interest.

## Figures and Tables

**Table 1:** Patient characteristics of the total collective.

Patient Characteristics	*n* (%)
Age (years)	Median 59.09 ± 13.1 Standard Deviation
Tumor foci	Unifocal 173 (54.2) Multifocal 146 (45.7)
Histology	No special type (NST) 188 (61.4) Non-NST 118 (38.5)
Estrogen Receptor	Negative 45 (18.3) Positive 201 (81.7)
Progesterone Receptor	Negative 92 (37.4) Positive 154 (62.6)
Tumor grade	G1 or G2 165 (52.2) G3 151 (47.7)
pT	pT1 197 (64.3) pT2–pT4 109 (35.6)
pN	pN0 166 (54.2) pN1–pN3 140 (45.7)
pM	pM0 239 (78.1) pM1 67 (21.8)
Nuclear RXRα	Negative 124 (42.5) Positive 168 (57.5)
Cytoplasmic RXRα	Negative 122 (40.0) Positive 183 (60.0)

**Table 2:** High cytoplasmic versus nuclear RXRα expression in correlation to clinicopathological data.

Variables	Cytoplasmic RXRα		Nuclear RXRα	
	*p*	Correlation Coefficient	*p*	Correlation Coefficient
pT	0.497	−0.039	0.012 *	−0.143
pN	0.630	0.028	0.063	−0.107
Histology	0.610	0.029	0.558	−0.034
Grading	0.029 *	0.125	0.227	−0.069

Clinicopathologic data and RXRα expression were correlated to each other in pT and grading status using Spearman’s correlation analysis. Significant correlations are in bold and indicated by asterisks (* *p* < 0.05) (*p*: two-tailed significance).

**Table 3:** Multivariate Cox regression analysis of nuclear RXRα expression in relation to OS.

Variable	Coefficient	HR (95% CI)	*p* Value
RXRn > 0 Histology	−0.310 −0.003	0.733 (0.487–1.103) 0.997 (0.970–1.024)	0.136 0.821
Grading	0.004	1.004 (1.000–1.009)	0.056
pT	0.551	1.736 (1.500–2.008)	**0.001**
pN	0.015	1.015 (1.006–1.024)	**0.001**
Focality	−0.240	0.787 (0.599–1.033)	0.085
Estrogen Receptor	0.084	1.088 (0.643–1.841)	0.754
Progesterone Receptor	−0.082	0.921 (0.545–1.557)	0.759

Significant results are shown in bold (*p* < 0.05); HR: hazard ratio; CI: confidence interval.

**Table 4:** Multivariate Cox regression analysis of cytoplasmic RXRα expression in relation to OS.

Variable	Coefficient	HR (95% CI)	*p* Value
RXRc > 0 Histology	0.472 −0.003	1.603 (0.964–2.665) 0.997 (0.973–1.023	0.069 0.837
Grading	0.004	1.004 (0.999–1.009)	0.132
pT	0.572	1.772 (1.514–2.074)	**0.001**
pN	0.013	1.013 (1.003–1.023)	**0.010**
Focality	−0.172	0.842 (0.623–1.139)	0.265
Estrogen Receptor	0.253	1.288 (0.725–2.291)	0.253
Progesterone Receptor	−0.254	0.776 (0.437–1.377)	0.386

Significant results are shown in bold (*p* < 0.05); HR: hazard ratio; CI: confidence interval.

**Table 5:** Multivariate Cox regression analysis of cytoplasmic RXRα expression in relation to DFS.

Variable	Coefficient	HR (95% CI)	*p* Value
RXRc > 0	0.528	1.696 (1.077–2.671)	**0.023**
Histology	−0.059	0.942 (0.873–1.017)	0.127
Grading	−0.001	0.999 (0.995–1.004)	0.811
pT	0.353	1.423 (1.190–1.701)	**0.001**
pN	0.000	1.000 (0.986–1.014)	0.996
Focality	0.040	1.041 (0.782–1.386)	0.783
Estrogen Receptor	−0.173	0.841 (0.488–1.451)	0.534
Progesterone Receptor	0.172	1.188 (0.689–2.047)	0.535

Significant results are shown in bold (*p* < 0.05); HR: hazard ratio; CI: confidence interval.

## Data Availability

The data presented in this study are available on request from the corresponding author. The data are not publicly available due to ethical issues.

## Abbreviations

BC	Breast cancer
DFS	Disease-free survival
NR	Nuclear receptor
OS	Overall survival
IRS	Immmunoreactive score
ER	Estrogen receptor
PBS	Phosphate-buffered saline
PR	Progesterone receptor
RXR	Retinoid X receptor
RXRα	Retinoid X receptor alpha
RXRɣ	Retinoid X receptor gamma
TC	Total collective
VDR	Vitamin D receptor
THR	Thyroid hormone receptor
